# Effect of Antiprogesterone RU486 on VEGF Expression and Blood Vessel Remodeling on Ovarian Follicles before Ovulation

**DOI:** 10.1371/journal.pone.0095910

**Published:** 2014-04-22

**Authors:** Annunziata Mauro, Alessandra Martelli, Paolo Berardinelli, Valentina Russo, Nicola Bernabò, Oriana Di Giacinto, Mauro Mattioli, Barbara Barboni

**Affiliations:** Faculty of Veterinary Medicine, University of Teramo, Teramo, Italy; Queen’s University, Canada

## Abstract

**Background:**

The success of ovarian follicle growth and ovulation is strictly related to the development of an adequate blood vessel network required to sustain the proliferative and endocrine functions of the follicular cells. Even if the Vascular Endothelial Growth Factor (VEGF) drives angiogenesis before ovulation, the local role exerted by Progesterone (P_4_) remains to be clarified, in particular when its concentration rapidly increases before ovulation.

**Aim:**

This *in vivo* study was designed to clarify the effect promoted by a P_4_ receptor antagonist, RU486, on VEGF expression and follicular angiogenesis before ovulation, in particular, during the transition from pre to periovulatory follicles induced by human Chorionic Gonadotropins (hCG) administration.

**Material and Methods:**

Preovulatory follicle growth and ovulation were pharmacologically induced in prepubertal gilts by combining equine Chorionic Gonadotropins (eCG) and hCG used in the presence or absence of RU486. The effects on VEGF expression were analyzed using biochemical and immunohistochemical studies, either on granulosa or on theca layers of follicles isolated few hours before ovulation. This angiogenic factor was also correlated to follicular morphology and to blood vessels architecture.

**Results and Conclusions:**

VEGF production, blood vessel network and follicle remodeling were impaired by RU486 treatment, even if the cause-effect correlation remains to be clarified. The P_4_ antagonist strongly down-regulated theca VEGF expression, thus, preventing most of the angiogenic follicle response induced by hCG. RU486-treated follicles displayed a reduced vascular area, a lower rate of endothelial cell proliferation and a reduced recruitment of perivascular mural cells. These data provide important insights on the biological role of RU486 and, indirectly, on steroid hormones during periovulatory follicular phase. In addition, an *in vivo* model is proposed to evaluate how periovulatory follicular angiogenesis may affect the functionality of the corpus luteum (CL) and the success of pregnancy.

## Introduction

Dominant preovulatory follicles are selected to grow from a pool of antral follicles during the ovarian cycle. This process leads to ovulation and CL formation [1]–[4]. Follicle selection success is strictly related to the development of a widespread blood vessel network required to sustain the enhanced proliferative and endocrine function of follicles [5]–[12]. Blood vessels allow growing follicles to acquire an increasing amount of nutrients, precursors, and hormones, as to release steroids and other regulating ovarian hormonal molecules to the systemic circulation [8], [13]. Several factors drive follicle angiogenesis indirectly controlling ovarian follicle development [6], [7], [11], [14]–[16]. VEGF is recognized to be a key molecule [13], [16], [17]. Indeed, its increased secretion, in addition to augmented vascular extension, is a necessary condition for large preantral follicles progression toward the antral stage [11], [18], [19]. Similarly, VEGF is up regulated in dominant follicle/s selection process leading to ovulation [7], [20]–[22]. Conversely, the process of follicle atresia is characterized both by VEGF and follicular blood vessel network reduction [14], [23]–[26].

VEGF, during gonadotropin surge, controls the crucial follicles transition from preovulatory to periovulatory stage that precedes ovulation [12], [16], [27]. In this time period of 24 or 44 h, depending on species, follicle profoundly changes its morphology and function [1], [25]. Luteinizing hormone (LH) surge induces a progressive disorganization of follicle basal membrane caused by proteolityc enzymes activation. Moreover, it modifies the steroid enzymatic pathway transforming an estrogen secreting preovulatory follicle to a P_4_ producing periovulatory follicle [28], [29]. In addition, follicular blood vessels undergo to dramatic changes. For the first time, large blood vessels appear in follicle walls and a higher blood flow is recorded [6], [7], [10], [27], [30]. Experimental evidences show that VEGF controls this vascular remodeling [10], [11], [21], [22], [27]. Indeed, its inhibition stops endothelial cell proliferation, impairs follicle angiogenesis preventing preovulatory follicle growth and ovulation [17], [22], [31], [32]. Although VEGF role on follicle development is clearly established, the mechanisms involved in its local expression remain to be clarified. Gonadotropins seem to link follicle growth and blood vessel remodeling through VEGF expression regulation [6], [7], [27], [33], [34]. However, it remains to be clarified whether gonadotropins directly affect VEGF expression or if this angiogenic factor is indirectly influenced by follicular steroidogenesis, as suggested by *in* *vitro* [33], [35] and *in vivo* experiments [36]. Amongst steroids, P_4_ may have a role in inducing VEGF expression [37], [38], endothelial cell proliferation [39]–[41], and angiogenesis [19], [42]. Indeed, the administration of P_4_ antagonist molecule, RU486, significantly decreased VEGF synthesis *in vivo*, in rat ovary [43] and in monkey endometrium [37]. Even if P_4_ influence on ovarian blood flow was demonstrated [44]–[47], during the periovulatory stage, when the LH surge induces a complete inversion of follicular steroid secretion [8], the role exerted by steroids on follicular VEGF synthesis still remains to be investigated.

To this aim, the present *in vivo* study was designed to study the effects induced by P_4_ antagonist administration, RU486 [48]–[53], on VEGF expression and angiogenesis during the transition from preovulatory to periovulatory follicles in gilts. This phase of follicle development was *in vivo* reproduced using a validated hormonal protocol [7], [27] able to promote antral follicular growth until the preovulatory stage (eCG injection) and ovulation (hCG treatment). Granulosa and theca VEGF contents were studied in selected follicles and its expression was correlated with follicle morphology and blood vessel organization assessed through histological, immunofluorescence and biochemical studies.

## Materials and Methods

### Ethical Committee

The experiments were approved by the Ethics Committee of the Universities of Teramo and Chieti-Pescara (Prot. 81/2011/CEISA/COM). All surgery was performed under sodium pentobarbital anesthesia, and all efforts were made to minimize suffering.

### Animal Treatments and Ovary Collection

**Figure 1:**
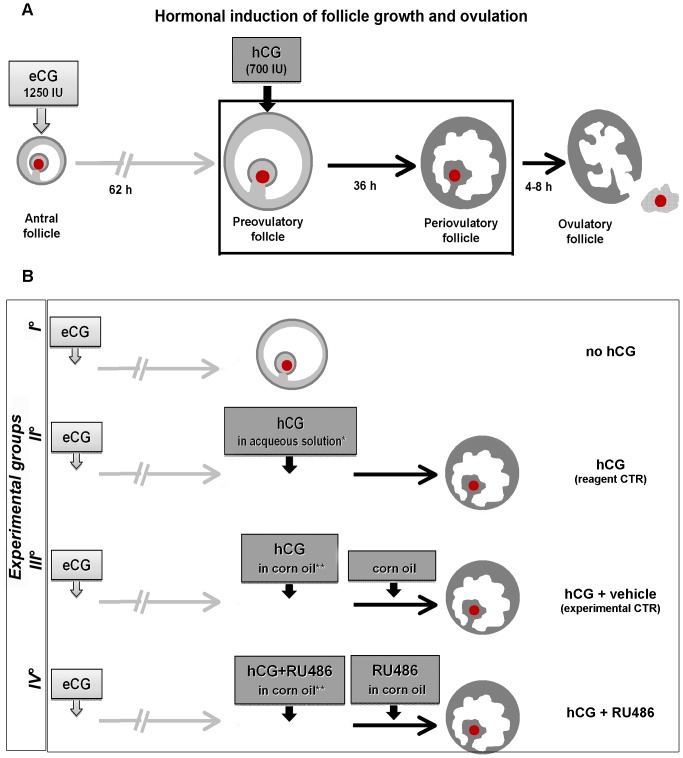
Experimental Plan. A) A schematic illustration of *in vivo* hormonal synchronization able to promote follicle growth and ovulation in prepubertal gilts. The periovulatory interval studied is showed in the box. B) Different experimental treatments and animal groups. All groups (n = 5 animals for each group) received eCG injection. Sixty-two hours after the eCG treatment, the first group (I°) was ovariectomized to obtain preovulatory follicles (no hCG), while the remaining groups received hCG treatment, with or without RU486. In detail, the second group (II°) received hCG injection dissolved in aqueous solution (*), as reagent control (CTR); the third group (III°) received hCG dissolved in corn oil (**), as experimental CTR, followed by administration of a corn oil alone 18 hours later; the fourth group (IV°) received hCG in combination with RU486 dissolved in corn oil and, 18 hours later, a second RU486 injection. Few hours before ovulation (36 hours after hCG treatments) the animals of these experimental groups were ovariectomized to obtain hCG, hCG+vehicle and hCG+RU486 periovulatory follicles. *commercial solvent **solvent for RU486.

Follicular growth and ovulation were pharmacologically promoted into 20 prepubertal Large White gilts with a mean weight of 90.7±1.16 Kg (mean ± Standard Error, SE) using two sequentially intramuscular (i.m.) administrations of 1250 IU of eCG (Folligon; Intervet International B.V.-Boxmeer, Netherlands) and 750 IU of hCG (Corulon; Intervet International-Boxmeer, Netherlands), respectively [6], [7], [27]. The animals were divided in four experimental groups (5 animals each) as summarized in Fig. 1. Ovariectomies [27] were performed in order to collect preovulatory follicles 62 hours after eCG treatment (no hCG) and periovulatory ones collected 36 hours after hCG injection (Fig. 1B).

In order to investigate the RU486 (Mifepristone, Sigma, St. Louis, USA) [48], [51] effects on periovulatory follicles, the hCG injection was carried out in combination with RU486. In particular, RU486 was solubilized in corn oil (10 ml) and i.m. injected at a concentration of 10 mg/kg [48]. RU486 half-life span is about 18–20 hours [51], [54], thus, two consecutive RU486 administrations were carried out: the first one in combination with hCG, the second one, 18 hours later, in corn oil (hCG+RU486) (Fig. 1B). The controls (CTR) were performed administrating hCG solubilized in aqueous solution (hCG, reagent CTR) or in corn oil (hCG+vehicle, experimental CTR) (Fig. 1B).

### Sample Preparation

After ovariectomy, one ovary was fixed in 4% paraformaldehyde/phosphate-buffered saline (PBS; pH 7.4) for 12 h at 4°C, dehydrated and embedded in paraffin wax before performing histological and immunohistochemical analyses. The contra lateral ovary was collected to isolate single healthy preovulatory (8 mm<diameter ≥7 mm) and periovulatory follicles (11 mm ≤ diameter >8 mm), as previously reported [6], [27]. Each single follicle was opened under a stereomicroscope to collect follicular fluid (FF) and isolate follicular wall. Each follicular wall was dissected in order to separate granulosa cells and theca shell [6], [7], [27]. In detail, the follicle wall obtained from each structure was transferred into dissection medium and, with the aid of a small spatula, the granulosa layer was gently scraped away from the theca shell. The medium containing dispersed granulosa cells was collected and centrifuged, while the theca shell was vigorously vortexed and carefully washed in order to remove any possible granulosa cell contamination (as preliminary demonstrated by histological analysis) [6]. FF, granulosa and theca samples were individually stored in liquid nitrogen.

### Morphological and Morphometric Analyses of the Follicular Response

Paraffin sections of 5 µm of thickness were serially collected on poly-L-lysine-coated slides before processing them for morphological, immunohistochemical (IHC) and morphometric investigations, according to Martelli et al. [11], [12], [27]. More in detail, at least three different sections/follicle were processed to analyze:

tissue architecture, follicular diameter and oocyte’s health with Hematoxylin-Eosin (HE) staining;
endothelial cells localization and vascular area (VA) extension using von Willenbrand Factor (vWF) IHC detection [27];
VEGF distribution within granulosa and theca compartment using IHC detection [11];
endothelial cell proliferation using vWF and Ki-67 double IHC (dIHC) detection [11], [27];
blood vessel maturation and mural cell recruitment using vWF and alpha Smooth Muscle Actin (α-SMA) dIHC analysis [27]. The used antibodies are summarized in Table 1.

**Table 1:** Details of the antibodies used in IHC.

Primary Abs (Company details)		Secondary Abs (Company details)	
	µg/ml		µg/ml
**Ki67**	5	**Anti-Mouse Cy3**	2
(Abcam, Cambridge, UK)		(Sigma-Aldrich, Missouri, USA)	
**vWF**	0.02	**Anti-Rabbit FITC**	5
(Dako, Glostrup, Denmark)		(Sigma-Aldrich, Missouri, USA)	
**α-SMA**	4	**Anti-Mouse Alexa Fluor488**	5
(Abcam, Cambridge, UK)		(Sigma-Aldrich, Missouri, USA)	
**VEGF**	100	**Biotinylated Anti Rabbit** a	5
(Calbiochem, USA)		(Vector Lab, Burlingame, USA)	

a Vectastain ABC Kit – Diaminobenzidine (DAB) used for detection.

VA values were determined on the follicular wall (Tot VA) or on the inner and outer blood vessel network (iN and oN, respectively) by quantifying the relative vWF-positive area (µm^2^) determined within a defined field of 15000 µm^2^ [11], [12], [27]. The results were recorded on at least five different sections/follicle and expressed as mean values ± SE.

Endothelial cells proliferation index (PI) was expressed as the percentage of Ki67 positive endothelial cells (Ki-67-and vWF double-stained cells) on the total number of theca cells identified by 4′-6-diamidino-2-phenylindole dihydrochloride (DAPI) fluorescent nuclear staining [27].

Morphometric analyses were performed at x 400 Magnification according to Martelli et al. [11], [27] with an Axioscop-2plus-epifluorescence microscope (Zeiss) provided with an interactive and automatic image analyzer (Axiovision, Zeiss). Morphometrical analysis was then performed by using a KS300 computed image analysis system (Zeiss).

### VEGF Biochemical Analysis

The levels and distribution of VEGF protein were analyzed in FF, granulosa and theca compartments.

### VEGF Content in FF

VEGF content in FF samples was analyzed using a specific ELISA assay (Quantikine; R&D Systems, Minneapolis, MN) as previously described [6], [7], [27]. VEGF soluble levels recorded on at least five different samples of FF/hormonal treatment were expressed as ng/ml (mean values) ± SE.

### VEGF Protein Content in Granulosa and Theca Compartments

Proteins extracted from granulosa or theca layers of each single isolated follicle (n = 5 follicles/gilt/treatment) were processed according to Martelli et al. [27]. Proteins (75 µg) were separated by 12% SDS-PAGE and electrophoretically transferred into a nitrocellulose membrane (Hybon C Extra; Amersham Pharmacia, Piscataway, NJ, USA) for Western Blot analysis [55]. Protein detection was performed by incubating the membranes with the primary polyclonal anti-human VEGF-antibody (2.5 µg/ml); Calbiochem, La Jolla, CA). The goat anti-rabbit immunoglobulins peroxidase-conjugated (IgG-HRP; 0.1 µg/ml, Santa Cruz Biotechnology Inc, Heidelberg, Germany) were finally used as secondary antibody. The membranes were re-probed with a monoclonal anti α-Tubulin (2 µg/ml, Sigma, St Louis, USA) and a goat anti-mouse IgG-HRP secondary antibody (0.1 µg/ml, Santa Cruz Biotechnology Inc, Heidelberg, Germany). The signals were detected with ECL Western Blot analysis system (Amersham Pharmacia, Amersham Pharmacia, Piscataway, NJ, USA). VEGF protein expression quantitative data were determined as the mean ratio of the optical density of specific bands normalized for the α-Tubulin expression through Advanced Image Data Analyzer (Rai Test; GMBH, Straubenhardt, Germany).

### VEGF mRNA Content Recorded In Granulosa and Theca Layers Using Real Time PCR

Total RNA was extracted from granulosa and theca tissues of each single isolated follicle using TRI-Reagent (Sigma, S Louis, USA) as previously described [56]. Purified RNA was re-suspended in RNAse- free water and spectrophotometrically quantified (A_260 nm_). One µg of RNA was electrophoretically separated in 1% Agarose gel in order to determine RNA quality. The RNA was treated with DNase I digestion (Sigma) for 15 min at room temperature. One µg of total RNA was used for reverse transcription reaction with Oligo dT primer and BioScript™ (Bioline, London, UK).

Real-time quantitative PCR was performed with Stratagene Mx3000P instrument, (Stratagene, La Jolla, CA) using SYBR Green I dye detection accordingly to Barboni et al [56]. Detailed information on *VEGF* and *β-Actin* [57] gene primers, product length and cycles are reported in Table 2. The reaction components were prepared at a concentration of: 2.5 µl forward (0.25 µM) primer, 2.5 µl reverse (0.25 µM) primer (Table 2), 3.5 µl water and 12.5 µl Brilliant SYBR Green QPCR Master Mix 2X (Stratagene, La Jolla, CA). Three µl of cDNA were added to 22 µl of master mix. The real-time protocol used was: denaturation for 10 min at 95°C, 45 cycles at 95°C for 1 min, annealing at 60°C for 1 min, extension at 72°C for 1 min. Each sample/follicle was run in triplicate, and normalized to the housekeeping *β-Actin* gene mRNA level. The amplicons specificity was confirmed by dissociation curve and by 2% agarose gel electrophoresis. Each sample was run in triplicate and for quantitation of *VEGF* gene target the Comparative Ct Method was applied for all samples normalized to the control housekeeping *β-Actin* gene using the formula: 2^−ΔΔC(t ) = 2 −(ΔCt control gene − ΔCt target gene)^. Statistical analysis was performed considering the last 3 experiments with a mean of 5 follicles/gilts/experiment.

**Table 2:** Primer sequences used for Real Time PCR.

Gene	Accession No.	Primer sequences	Size (bp)	Cycles
***β-ACTIN*** *	U07786*	F: ATCGTGCGGGACATCAAGGA*	178	40
	Bos Taurus	R: AGGAAGGAGGGCTGGAAGAG*		
***VEGF***	X81380	F: GAAGTGGTGAAGTTCATGGA	299	40
	Sus Scrofa	R: GCCTTGCAACGCGAGTCTGT		

*primer sequences by Shimizu T et al. [57].

### Statistical Analysis

The data were expressed as mean ± SE of at least 3 independent experiments. The quantitative data obtained from the different hormonal treatments were, firstly assessed for normalcy of distribution, by D′Agostino and Pearson test. Then, the data sets were compared using ANOVA test followed, when necessary, by post-hoc Tukey test (GraphPad Prism 5, GraphPad Software, USA). The data were considered significant for *p*≤0.05.

## Results

### Ovarian Response to Hormonal Treatment

Macroscopic ovary evaluation showed that preovulatory and periovulatory follicular rate was not influenced by RU486 treatments as summarized in Table 3. In detail, preovulatory follicles (no hCG) were 21.15±2.17/animal and periovulatory ones were 19.85±2.21 (*p*>0.05), 21.02±1.74 (*p*>0.05) and 18.96±1.62 (*p*>0.05) in hCG, hCG+vehicle and hCG+RU486 animals, respectively.

**Table 3:** Evaluation of preovulatory follicles (no hCG) and periovulatory follicles, 36 hours after hCG, with or without RU486, collected from hormonally-induced prepubertal gilts.

Hormonal Treatments	N. of follicles/animal	Follicle mean diameter (Ø)
no hCG	21.15±2.17	7.4±0.4 mm
hCG	19.85±2.21	9.6±1.4 mm
hCG+vehicle	21.02±1.74	9.6±1.4 mm
hCG+RU486	18.96±1.62	9.6±1.4 mm

Statistically not different values, *p*>0.05.

Values represent mean ± SE.

**Figure 2:**
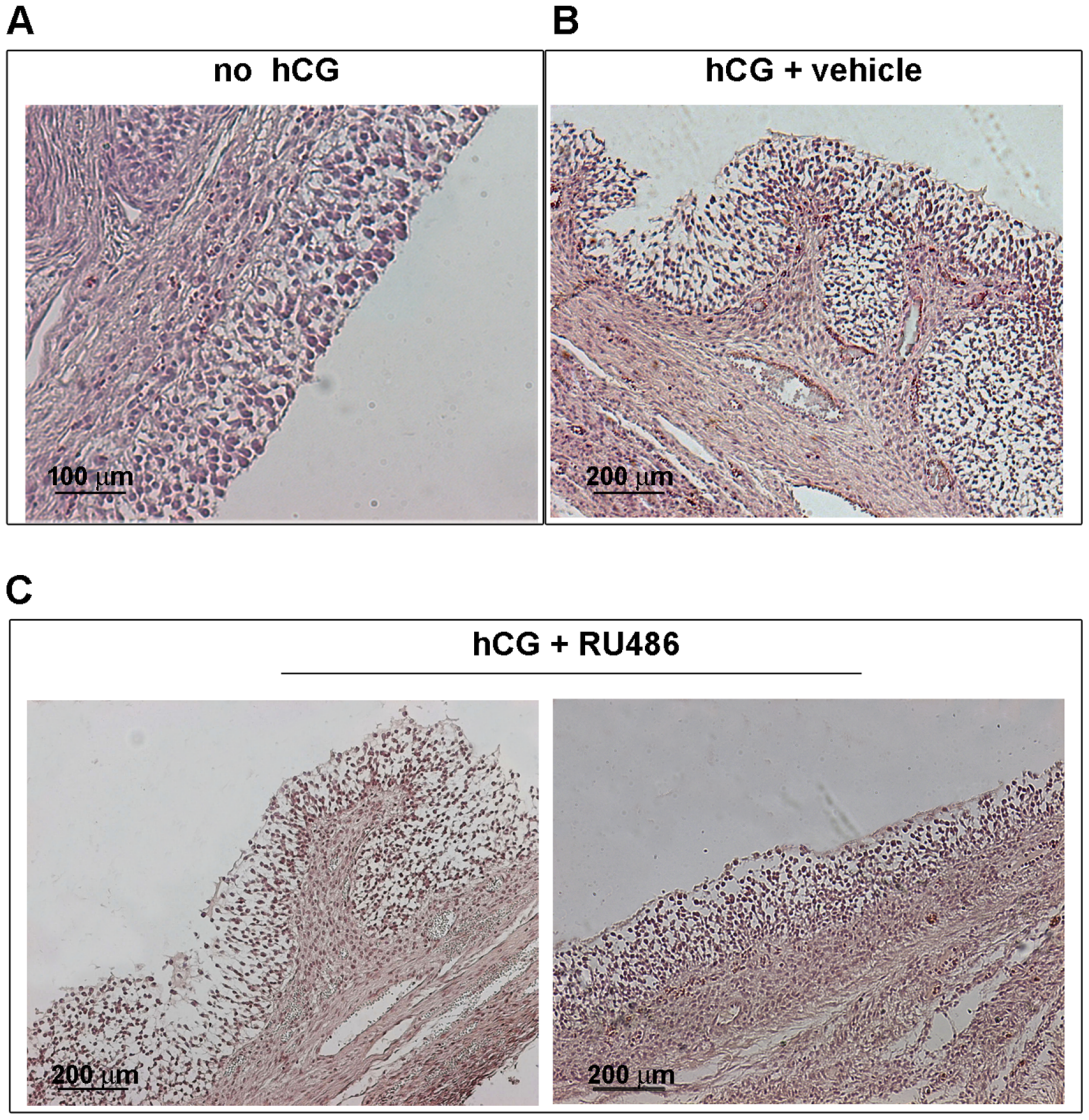
Ovarian follicles morphology subsequent to different hormonal treatments. Representative follicle images of tissue sections stained with HE. A) Preovulatory follicles (no hCG); B) hCG+vehicle periovulatory follicles; C) hCG+RU486 periovulatory follicles: left image, an example of a follicle isolated after RU486 treatment that displayed a typical periovulatory structures; right image, an example of a follicle isolated after RU486 treatment that showed a preovulatory-like morphology.

HE staining (Fig. 2) confirmed that all isolated follicles were healthy and contained an oocyte surrounded by a cumulus cells continuous layer. They displayed a nucleus with a condensed chromatin and an uniform cytoplasm appearance (data non shown). Preovulatory follicles (n = 24; no hCG) showed a microscopic spherical regular architecture with a compact mural granulosa layer apposed on the basal membrane (Fig. 2A). Differently, periovulatory follicles isolated after hCG (data not shown) or hCG+vehicle (Fig, 2B) treatments displayed a typical dispersed granulosa layer, with infoldings of the theca projected towards the antrum. No vehicle effect was observed on periovulatory follicles. This periovulatory morphology was observed only in ∼ 30% of follicles isolated from RU486 treated gilts (n = 28; Fig, 2C, left image), while the remaining 70% maintained a structural organization that was similar to preovulatory follicles (Fig. 2C, right image).

### VEGF Protein Content-in Follicular Fluid and Granulosa/theca Compartments

**Figure 3:**
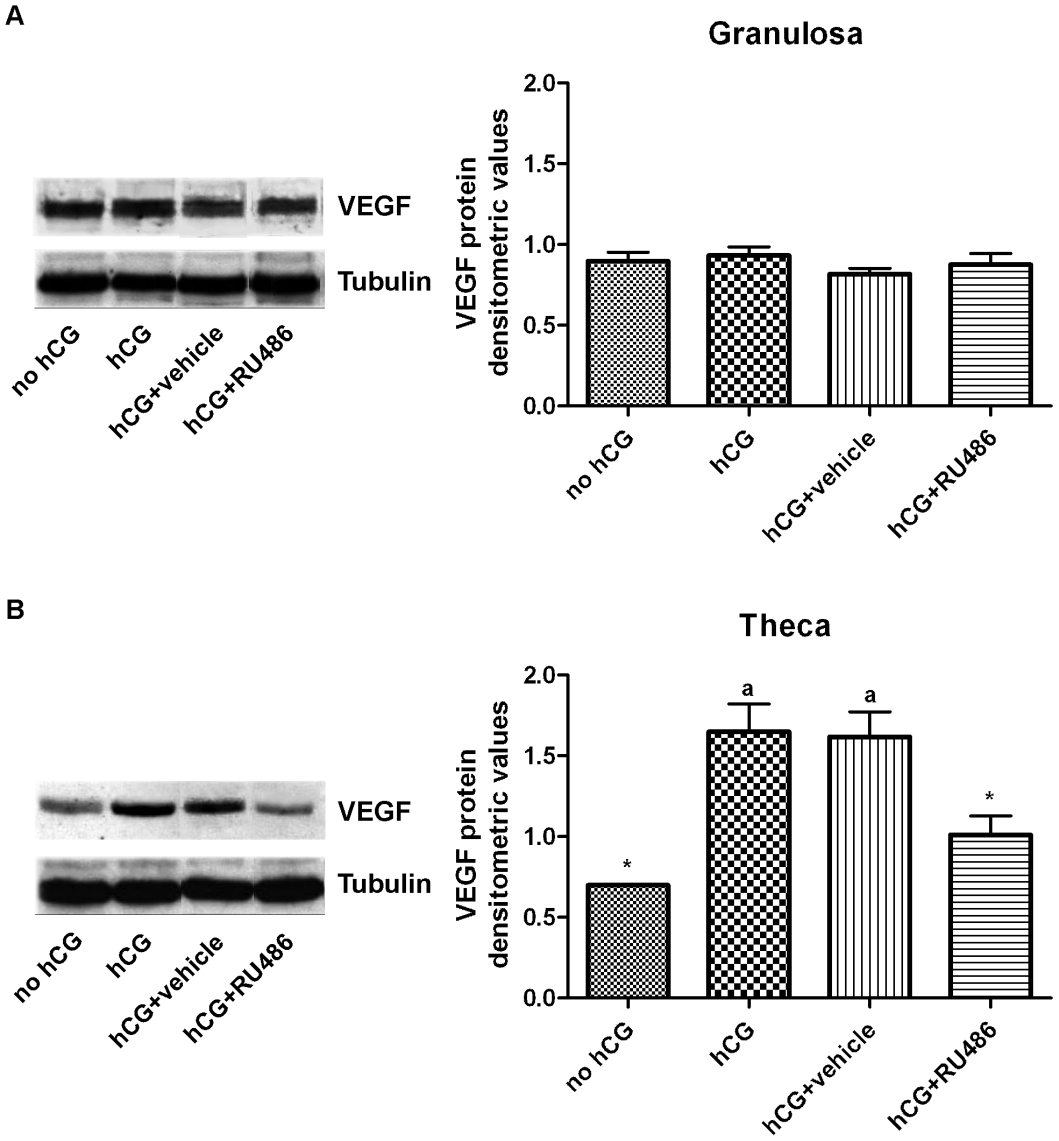
VEGF protein content recorded in granulosa and theca compartments of preovulatory and periovulatory follicles. Representative Western Blot images of VEGF_164_ protein expression in A) granulosa cells and B) theca compartment of preovulatory follicles (no hCG), and hCG, hCG+vehicle, or hCG+RU486 periovulatory follicles. The histograms indicate VEGF_164_ densitometric values normalized for α-Tubulin. The values are expressed as mean ± SE of 3 independent experiments for a total of 15 follicles/treatment. ^a^ significantly different values (*p*<0.05) *vs.* no hCG treatment * significantly different values (*p*<0.05) *vs.* hCG+vehicle treatment.

FF, granulosa cells and theca layer were separately collected by each follicle with the aid of a stereomicroscope. As summarized in Table 4, VEGF content in FF was 15.96±0.53 ng/ml in preovulatory follicles (no hCG), resulted undetectable after both hCG treatments (hormone alone or solubilized in vehicle), and significantly lower in RU486 periovulatory follicles (0.98±0.07 ng/ml; Table 4). Granulosa VEGF protein expression, was similar in all analyzed preovulatory and periovulatory follicles (Fig. 3A). Instead, theca VEGF levels increased in both periovulatory hCG and hCG+vehicle follicles (Fig. 3B), while it did not change in hCG+RU486 follicles and it resulted similar to those recorded in preovulatory ones (Fig. 3B).

**Table 4:** VEGF protein content in follicular fluid (FF) of preovulatory (no hCG) and periovulatory follicles, 36 hours after hCG, with or without RU486, collected from hormonally-induced prepubertal gilts.

Hormonal Treatments	Number of follicles	VEGF ng/ml (mean ± SE)
no hCG	24	15.96±0.45
hCG	28	n.d
hCG+vehicle	25	n.d
hCG+RU486	28	0.98±0.07^a^

n.d: undetectable values.

a statistically different values (*p*<0.01) *vs.* no hCG.

### VEGF Protein Distribution in Follicular Compartments

**Figure 4:**
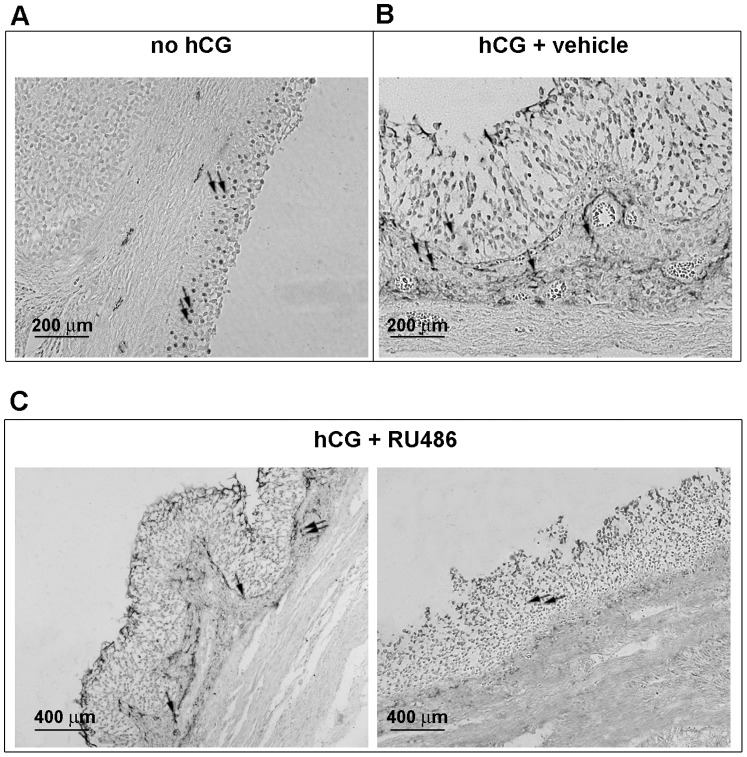
VEGF protein distribution within preovulatory and periovulatory follicles collected after different treatments. Representative follicle images displaying VEGF protein localization detected by IHC. VEGF was revealed by black positivity (DAB signal) detected within cells (black arrows) or deposited amongst the extracellular matrix. A) preovulatory follicle (no hCG), B) hCG+vehicle periovulatory follicle, and C) hCG+RU486 follicle: left image, an example of RU486 follicle that displayed a typical periovulatory structure; right image, an example of RU486 follicle that showed a preovulatory-like morphology.

VEGF protein distribution within granulosa and theca layers was investigated using IHC, as shown in Fig. 4. In all categories of follicles, VEGF was present within several granulosa cells or accumulated amongst them. By contrast, VEGF localization in theca compartment resulted influenced by hormonal treatments. More in detail, in preovulatory follicles (no hCG, Fig. 4A) VEGF was exclusively localized near the blood vessels. Differently, both periovulatory hCG (data not shown) and hCG+vehicle follicles (Fig. 4B) showed a preferential extracellular matrix VEGF distribution close to the basal membrane and to blood vessels. A similar theca VEGF distribution was observed in RU486 periovulatory follicles (Fig. 4C), even if in those follicles that maintained a preovulatory-like organization its distribution close to the blood vessels appeared lower, if not absent (Fig. 4C, right image).

### *VEGF* mRNA Expression in Granulosa and Theca Layers

**Figure 5:**
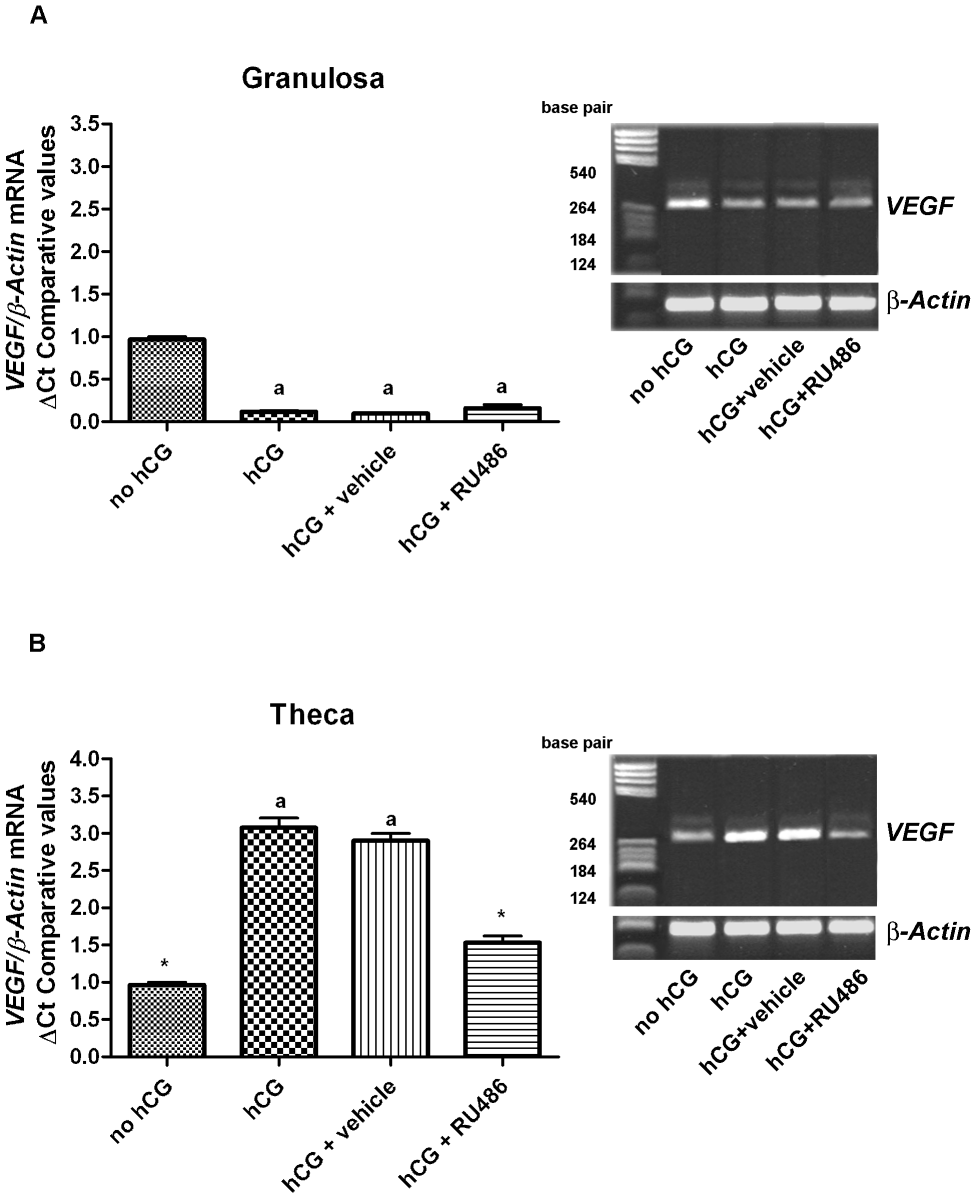
Quantitative *VEGF* mRNA expression recorded in granulosa and theca compartments of pre and periovulatory follicles. Representative images displaying *VEGF*_164_ mRNA expression analyzed by Real Time PCR. A) granulosa and B) theca compartment of preovulatory follicles (no hCG) and hCG, hCG+vehicle, or hCG+RU486 periovulatory follicles. The histogram indicates *VEGF* values quantified by a Comparative C_t_ method and expressed as the mean ± SE of 3 independent experiments for a total of 15 follicles/treatment. Amplicons specificity was confirmed by 2% agarose gel electrophoresis. ^a^significantly different values (*p*<0.05) *vs.* no hCG treatment. *significantly different values (*p*<0.05) *vs.* hCG+vehicle treatment.

As shown in Fig. 5, *VEGF* mRNA expression significantly decreased in granulosa cells after hCG injection, regardless of the treatments used (Fig. 5A). Differently, *VEGF* mRNA levels increased in theca layer after hCG injection alone or with vehicle (Fig. 5B). By contrast, *VEGF* up regulation was absent in theca layers of follicles obtained from RU486 treated animals (Fig. 5B, hCG+RU486 *vs* hCG+vehicle, *p*≤0.05) and its level remained similar to those of preovulatory ones (Fig. 5B, hCG+RU486 *vs.* no hCG, *p*≥0.05).

### Blood Vessel Remodeling during RU486 Treatment

**Figure 6:**
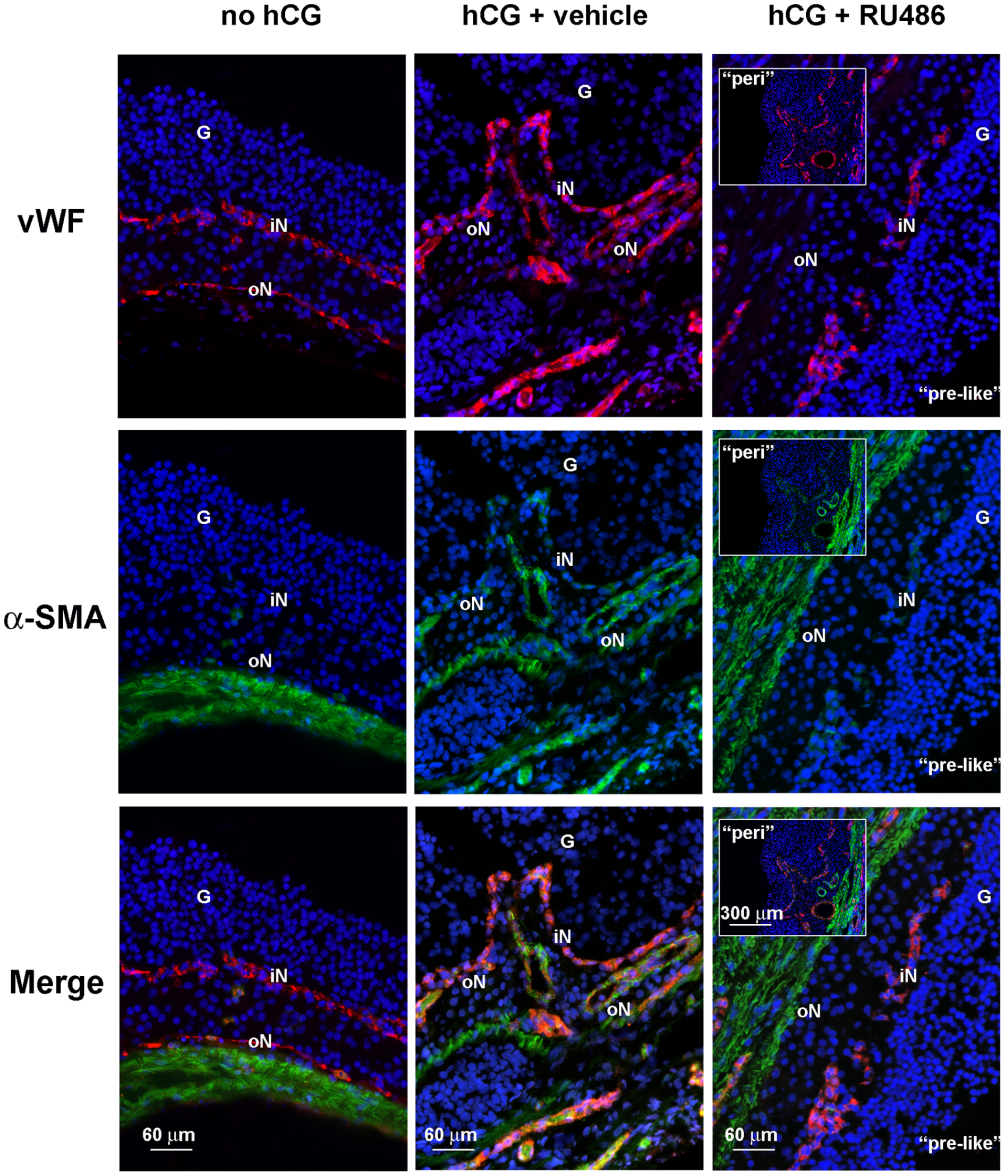
vWF and α-SMA protein distribution in preovulatory and periovulatory follicles. Representative images of double IHC performed on preovulatory (no hCG) and hCG+vehicle or hCG+RU486 periovulatory follicles to reveal the endothelial marker vWF (red fluorescence, Cy3) and, the smooth muscle actin (α-SMA) antigen (green fluorescence, Alexa Fluor 488). Cell nuclei were counterstained with DAPI (blue fluorescence) to easily distinguish granulosa (G), iN, and oN blood vessel networks localized in theca compartment. In hCG+RU486 image it is shown an example of the α-SMA and vWF distribution in a preovulatory-like organized follicle (“pre-like”), while in the insert panels RU486 examples are shown with the typical periovulatory morphology (“peri” in the left upper corner).

**Figure 7:**
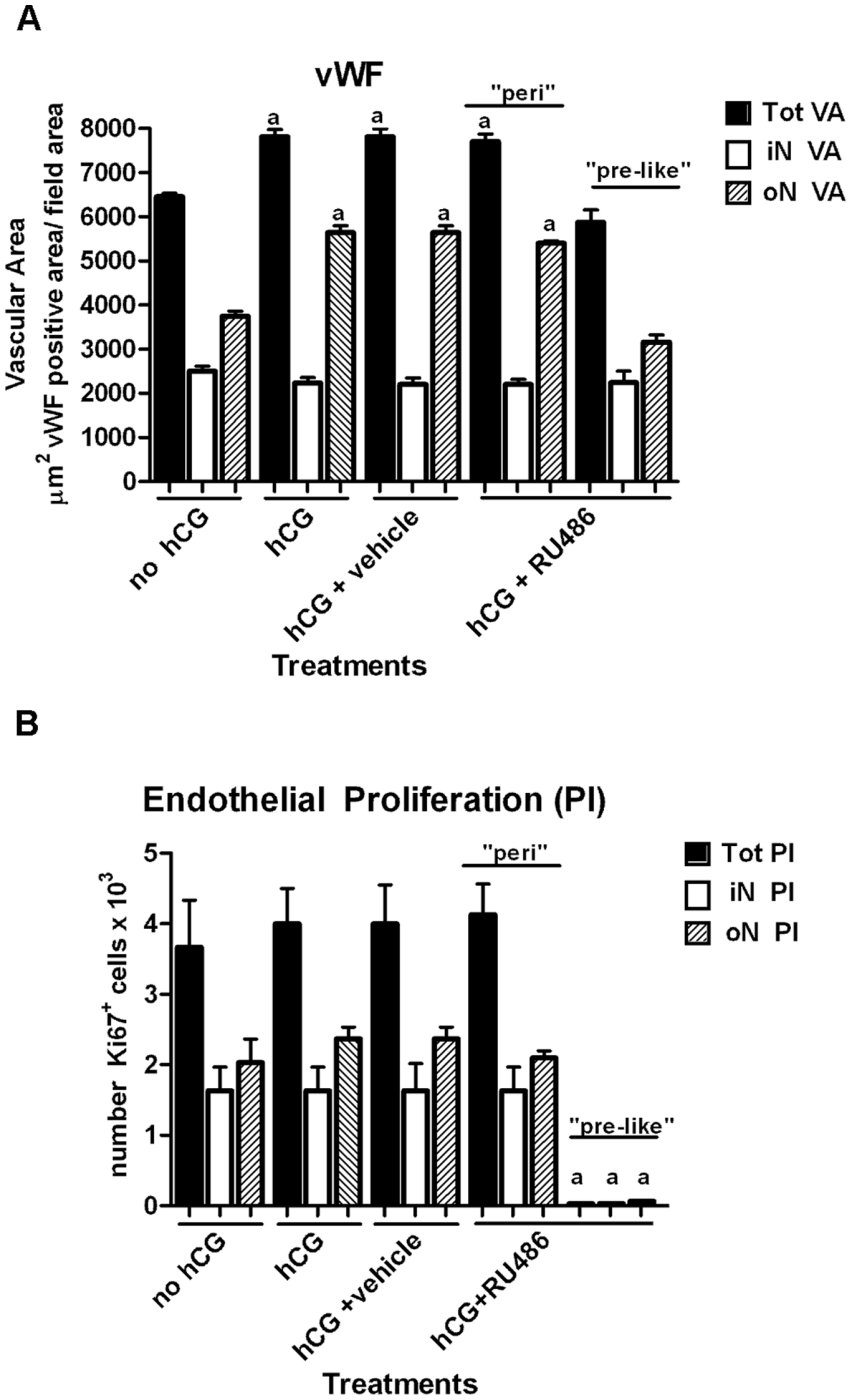
Vascular area and endothelial cell proliferation in pre and periovulatory follicles. A) The histogram shows the VA values calculated, in preovulatory follicles (no hCG), and in hCG, hCG+vehicle or hCG+RU486 periovulatory follicles, considering the vWF-positive area*/*15000 µm^2^ of theca field. The VAs were divided in iN (open bars) and oN (shaded bars), on the basis of their localization. The total VA (solid bars) expressed the iN VA+oN VA. The preovulatory–like (“pre-like”) and periovulatory (“peri”) hCG+RU486 follicle VAs were calculated separately. The data expressed as mean values ± SE were obtained from 15 different follicles/treatment. ^a^statistically different values (*p*<0.05) of each VA *vs.* no hCG preovulatory follicles. B) The histogram shows the endothelial cell PI given, after dIHC analyses, by the percentage of Ki 67 (a cell proliferation marker) positive cells that co-localize with the endothelial cells (anti-vWF marker) recorded in a standardized field (15.000 µm^2^ of theca layer field). Total PI (solid bars) was divided in iN (open bars) and oN (shaded bars) PIs, on the basis of their localization. The data expressed as mean values ± SE were obtained from 15 different follicles/treatment. ^a^statistically different values (*p*<0.01) of each PI *vs.* no hCG preovulatory follicles.

The effects of RU486 on blood vessel organization (Fig. 6), VA and endothelial cell proliferation (Fig. 7) were evaluated using IHC. Preovulatory follicles (no hCG) displayed a blood vessel network organized into two concentric structures connected to each other by anastomotic vessels. The iN VA (2661.82±89.46 µm^2^/field, Fig. 7A), characterized by capillaries close to the basal membrane (Fig. 6), was lower than the oN VA (3751.07±89.44 µm^2^/field, Fig. 7A). The Tot VA significantly increased after both hCG and hCG+vehicle treatments (Fig. 7A, *p*≤0.01). In particular, the oN VA dramatically increased (Fig. 7A) becoming ∼70% of Tot VA for the presence of new large blood vessels (Fig. 6).

Total and oN VAs similarly increased in follicles that acquired after RU486 treatment a periovulatory organization (Fig. 6 insert image and Fig. 7A, “peri”). On the contrary, VAs did not change in those follicles that maintained a preovulatory-like architecture which showed few large vessels within the theca (Fig. 6 and Fig. 7A, “pre-like”).

The vWF (red stain) and α-SMA (green stain) double-immunolabeling, performed to describe blood vessel maturity and perivascular recruited cells, showed a clear RU486 treatment effect. (no hCG) Blood vessels preovulatory follicles (iN and oN) did not display α-SMA immunopositivity (Fig. 6). Instead, perivascular α-SMA positive cells appeared in iN and oN of periovulatory follicles obtained after hCG (data not shown) and hCG+vehicle treatments (Fig. 6). In particular, the iN displayed an α-SMA immunopositivity close to the endothelial cells (vWF positive cells) forming a “chain-like” organization. By contrast, α-SMA positivity was observed at the periphery of the large blood vessel walls localized in oN (Fig. 6).

A similar distribution of α-SMA- positive cells was observed in those follicles isolated by RU486 treated animals that acquired a periovulatory organization (Fig. 6 insert image, “peri”). By contrast, the follicles maintaining the preovulatory-like architecture (Fig. 6, “pre-like”) did not display α-SMA positivity into the iN, and showed a reduced α-SMA perivascular immunoreaction in the oN large blood vessels. Rarely, these large blood vessels displayed a continuous α-SMA perivascular layer (Fig. 6, “pre-like”).

### vWF and Ki-67 Staining

The vWF and Ki-67 double immunolabeling was used to quantify the proliferating endothelial cells in the different classes of follicles (Fig. 7B). Preovulatory (no hCG) and periovulatory follicles (hCG and hCG+vehicle) displayed endothelial Ki-67 positive cells in the iN and oN. The incidence of proliferating endothelial cells significantly decreased only in follicles isolated from RU486 treated animals that maintaining the preovulatory-like architecture (Fig. 7B, “pre-like”).

## Discussion

The present study was performed to clarify the role of RU486 on VEGF-dependent ovarian angiogenesis that occurs *in vivo* in dominant follicles after hCG administration (to mimic LH surge). Our results demonstrate, for the first time, that VEGF production, blood vessel network, and follicle tissue remodeling were all affected by the *in vivo* administration of RU486 during the phase of transition from pre to periovulatory follicle, characterized by increasing levels of P_4_ [28], [29], even if the cause-effect correlation between these events remains to be clarified. In addition to pregnancy [52], [53], [58], [59], ovulation [60]–[65], and sperm-oocyte recognition [66], a new RU486 negative effect was demonstrated on reproductive events. Indeed, RU486 administration was able to impair periovulatory follicle development and angiogenesis induced by hCG. Its effect appeared to be compartment and vascular (oN and/or iN) network-dependent. In particular, RU486 treatment down-regulated VEGF expression within the theca compartment, while, it did not affect granulosa and FF VEGF content. Independently of RU486 injection, FF VEGF levels dramatically decreased after hCG treatment. This result could be apparently contradictory, since low FF VEGF levels were recorded both in RU486 and hGC periovulatory follicles characterized by low and high theca VEGF expression respectively. This observation, may be explained hypothesizing that hCG treatment, induces the synthesis of VEGF low bioavailability isoforms in follicles. This suggestion is in agreement with the expression of high molecular weight (i.e. VEGF_189_) VEGF isoforms with a lower solubility evidence in endometrium stimulated by a modified steroid milieu [67]. Alternatively, VEGF bioavailability could be affected by local changes of heparin or cell surface heparan sulphate concentration, as previously supposed by Robinson & Stringer [68]. Regardless the mechanism, RU486 was able to selectively inhibit VEGF expression, triggered by hCG, within theca compartment of periovulatory follicles. This inhibitory effect may be a consequence of the antiprogesterone action of RU486, as reported in bovine [69], human [70] and mink [71], and in another reproductive tissue such as endometrium [37], [38], [42], [72] exposed to low concentrations of P_4_. As a consequence, the RU486 follicles displayed an incomplete development of the blood vessel network. It is interesting to note as RU486 treatment did not influence the total VA but, specifically, affected oN VA that displayed a reduced extension. This important inhibitory RU486 influence was exclusively observed in 70% of the follicles that did not acquire the typical periovulatory organization. These results confirm that morphological and vascular remodeling recognize controlling cross-talk mechanisms [73]–[78]. On the contrary, RU486 treatment did not affect iN VA that probably remained regularly controlled by the unchanged FF and granulosa compartment VEGF levels. The presence of iN VA may have an essential functional role since it could maintain an adequate trophic supply of the germinal structure. Indeed, its persistence is vital to ensure the correct oxygen, precursors and metabolites supply either to the avascular granulosa compartment or to the oocyte [1], [6], [7], [27].

Though, the RU486 inhibitory effects described on endothelial cell proliferation and mural cell recruitment can not be exclusively explained with the theca VEGF down-regulation. Indeed, RU486 treatment induced these responses either the oN or iN. The inhibition of endothelial cell proliferation could be a consequence either of VEGF lower levels, accordingly to other Authors [75], [76], as of RU486 antagonist role on P_4_ receptor. This hypothesis seems to be supported by the observations of P_4_ receptors expression in Huvec cells [79], [80] and by the P_4_-dependent inhibition of endothelial cell proliferation, already demonstrated in endometrial blood vessels in mouse [42] and in uterine vasculature in pig [38]. RU486 inhibitory role on mural cell recruitment affected also the consequent blood vessel maturity. Human CG injection in periovulatory follicles promotes the maturation of blood vessels by recruiting mural cells (α–SMA positive cells). These cells in the oN surround large blood vessels, while in iN capillary network acquire the typical “chain-like organization” [27]. The RU486 negative influence on blood vessel maturity could be related to a direct effect of P_4_, as already suggested in mouse [42], or as a consequence of VEGF lower expression. It is known that this angiogenic factor indirectly modulates blood vessel maturation in other systems by influencing the secretion of key factors such as Angiopoietins/Tie-2 receptor [74], [75], [81], [82], platelet-derived growth factor (PDGF)-B/PDGF receptor beta [76], and the transforming growth factor beta (TGF beta) [76]. Regardless the mechanisms involved, blood vessels immaturity after RU486 treatment may impair the physiological transition from periovulatory follicle to CL structure. The presence of smooth muscle in follicle blood vessels is essential for the control exerted by nervous system or local hormones on the ovarian function [27]. In particular, the large vessels with a smooth muscle mural wall absence in the oN could probably impair blood flow required for ovulation and the subsequent CL development [83], [84]. Moreover, α–SMA positive cells absence within the capillaries of the iN could negatively affect blood vessel stabilization and haemodynamic processes necessary to support the early stage of CL growth [85]. In this context, further experiments will be required to demonstrate if and how the low maturity of follicular blood vessels could influence ovulation and CL function.

In conclusion, the obtained results in the present work demonstrate RU486 negative influences on VEGF expression, vascular and tissue remodeling, providing important insights on the biological *in vivo* role of this P_4_ antagonist during the transition from the preovulatory to the periovulatory stage. Even if the cause-effect correlation amongst angiogenesis and impaired follicle development has not been demonstrated, these results propose an *in vivo* model that allows to study the effect of an incomplete follicle maturation on female reproduction success.
